# Exploring patient participation during video consultations: A qualitative study

**DOI:** 10.1177/20552076231180682

**Published:** 2023-06-05

**Authors:** Martin Vinther Bavngaard, Elle Christine Lüchau, Elisabeth Assing Hvidt, Anette Grønning

**Affiliations:** 1 Department of Rehabilitation Science and Health Technology, Faculty of Health Sciences, Oslo Metropolitan University, Oslo, Norway; 2 Research Unit of General Practice, Department of Public Health, 6174 University of Southern Denmark, Odense, Denmark; 3 Department of Language, Culture, History and Communication, 6174 University of Southern Denmark, Odense, Denmark

**Keywords:** Digital health, qualitative, video consultations, patient participation, affordances, communication, telemedicine, general practice

## Abstract

**Objective:**

Video consultations enable a digital point of contact between the general practitioner and patient. With their medium-specific characteristics, video consultations may create novel conditions for the enactment of patient participation during consultations. Although numerous studies have explored patients’ experiences of video consultations, research explicitly investigating patient participation within this new consultation setting remains sparse. This qualitative study explores how patients participate during interactions with their general practitioner by drawing on the affordances of video consultations.

**Methods:**

The data corpus comprises eight recorded video consultations (59 minutes and 19 seconds in total) between patients and their general practitioner, all subjected to reflexive thematic analysis yielding three themes illustrating concrete participatory use cases.

**Results:**

We find that video consultations provide an accessible format for patients otherwise unable to attend a physical consultation due to physical and mental barriers. Moreover, patients participate by drawing on resources situated in their spatial setting to settle health-related questions of doubt arising during the consultation. Lastly, we posit that patients enact participation by visually communicating their impromptu engagement in decision-making and reporting to their general practitioner by making use of the qualities of their smartphone during their consultation.

**Conclusions:**

Our findings illustrate how video consultations provide a communicative context in which patients may enact distinct forms of participation by drawing on its technologically contingent affordances during interactions with their general practitioner. More research is needed to explore the participatory opportunities of video consultations in telemedical healthcare services for different patient groups.

## Introduction

In Denmark, consultations within general practice are primarily conducted in the clinic.^1^ At the same time, however, health authorities such as the Danish Health Data Authority and the Danish Ministry of Health have undertaken policy-driven efforts to digitalise the contact between healthcare professionals and patients, including in general practice,^2,3^ thus aiming to conduct one-third of all consultations in both general and hospital practice online.^4^ Moreover, the onset of the COVID-19 pandemic in 2020 sparked incentives directing general practitioners (GPs) and patients to use video consultations (VCs) to limit the risk of infection.^5^ Even in the wake of the first gradual reopening of the Danish society in April 2020, the continued use of non-physical consultation formats across all health sectors was strongly recommended by The Danish Health Authority.^6^

As a subdomain of telemedicine, VCs use ‘information and communication technology to deliver health care at a distance’.^7^^(p1)^ VCs have found use in a wide range of healthcare settings, including primary care, clinical oncology, specialist palliative and diabetes care and mental health therapy.^8^ Within general practice, VCs have – like email consultations^9^ – been found useful in mediating contact between GP and patient, not least because of their spatial flexibility.^10,11^ As a result of this flexibility, VCs connect the setting of general practice with that of the patient's self-selected geographical location,^12^ affording new ways for GPs and patients to participate in facilitating the consultation: patients are expected to download and set up the VC application themselves as well as secure a suitable physical place for the consultation to be held.^12,13^ Conversely, GPs may need to provide technical support for patients experiencing technical problems and discuss changing workflows induced by VCs within the healthcare team.^14^ As such, VCs create a context that calls on distinct forms of participatory action. Against this background, we seek to investigate the enactment of participation during VC-mediated interactions between patients and their GP.

Although *patient participation* coexists with a variety of similar concepts (e.g., patient involvement and patient engagement) related to the paradigm of patient-centred care,^15^ it is understood as concrete and observable actions by which patients take ‘active part in their consultation with professionals’.^16^^(p389)^ Participation may thus be enacted through both verbal and non-verbal means.^17,18^ Indeed, Street et al.^19^ mark non-verbal behaviour, such as eye contact and head nodding, as constituting patient participation and call for future investigations of such phenomena. To guide our study of how verbal and non-verbal participation may be enacted during VCs, we adopt the analytical perspective of Peräkylä et al.^20^ on patient participation as involving the concrete ways in which patients make use of opportunities to contribute to matters related to their health during the consultation.

Few studies have focused specifically on the patient when investigating the opportunities afforded by the VC as a medium, whether for participatory ends or not. Indeed, the theoretical concept *affordances*,^21^ denoting the specific use potentialities made possible by different technologies, has seen little use in research on VCs.^22^ In the context of product design, affordances are used to describe how an object may be perceived by an individual as affording certain uses. How a user may perceive and act upon an object depends on (1) the characteristics of the object and (2) the user's agentic use intentions as well as the user's preceding physical, cultural, and cognitive capacities.^23,24^ Conversely, the same capacities also determine *constraints* regarding use potentials: how an object may be perceived by an individual as *not* affording certain uses.^21^ To illustrate this point, we offer a deliberately hyperbolic example: to the individual able to navigate touchscreen interfaces and decode the meaning of contemporary applications and their various symbols, a smartphone may be utilised for browsing the web or video-calling distant relatives. To another, it might serve as an excellent paperweight simply due to its physical characteristics.

Although limited in quantity, some studies testify to the productivity of operationalising affordances to study participatory interactions during VCs. Stommel et al.^25^ identify visuality as an affordance inherent in the VC that allows patients to communicate bodily symptoms to the GP in real-time despite being physically separated. Similarly, Islind et al.^26^ illustrate how VCs afford an increased sense of intimacy when compared to physical consultations; for instance, by enabling patients to divulge sensitive information vital for their treatment. Although not overtly applying the concept of affordances, several studies present findings highly contingent on the medium-specific characteristics of VCs which imply certain use potentials actualised by patients for participatory ends - for instance, regarding what can and cannot be said during VCs when compared to telephone consultations.^27^ As found by Lüchau and Grønning,^28^ one patient may deem it inappropriate to talk about weight during a VC, while another may find it entirely unproblematic. Moreover, feelings of being more intimately connected to the GP through the VC format may give rise to more collaborative behaviour, heightening participation.^29,30^ Conversely, some patients perceive VCs as a more rigid format than physical consultations, thus restraining themselves from elaborating on health matters and effectively limiting their participation.^31^ Building upon this line of inquiry, and explicitly adopting the concepts of patient participation and affordances, this study addresses the following research question: *how do patients participate during their interaction with their GP by making use of the affordances of VCs?*

By focusing on the content of the VCs in the form of multimodal interactions (e.g., speech, gaze and gestures), we diverge methodically from most of the existing research that has similar aims, which mainly relies on interviews with patients as data. This allows us to investigate how patients interact with their GP *in situ* and thus to understand the practical implications of VCs for both patient communication and, by extension, participation. By generating new knowledge within this domain, our study contributes to the attunement of practitioners to communicative aspects of VCs which have often gone unnoticed. In this way, we seek to heighten the quality of the delivery of telemedical healthcare via VCs.

## Methods

The dataset for this case study consists of eight video-recorded VCs between patients and their GP, each lasting between 3m 19s and 20m 1s, amounting to a total of 59m 19s of footage. Data collection took place between May and December 2020, a period in which Denmark faced its first national lockdown during the COVID-19 pandemic. GPs were recruited through word of mouth within the authors’ professional network in the Region of Southern Denmark, one of Denmark's five geographically defined regions. Three GPs participated: two males and one female, connected to two clinics located in the same city.

Screen recording the VCs via the GP's office computer was deemed the most suitable data collection method, as the task of initiating and completing the recording could be performed by the GP single-handedly. This was an important methodological consideration, as having a researcher present during the consultation – either physically or digitally – could interfere with the natural dynamics of the interaction between patient and GP.^32^*Rec.vc* was chosen as the most expedient screen recording software programme, as it was compatible with the *My Doctor* app recommended by the Danish Organisation of General Practitioners (PLO) and through which all participating GPs conducted their VCs. Immediately upon completion, the recordings were transferred to a secure cloud service for external storage. At no time did participants (neither patients nor GPs) acquire access to the recordings.

Participating GPs provided informed written consent. All patients provided informed oral consent to having their VC recorded. The consent was given at the beginning of their VC, after which the recording commenced. After providing consent, all participants were presented with the option of allowing the research group to publish non-anonymised still images from the recorded VC as part of their research. Face blurring has been used to conceal the identity of those who declined. Subsequently, confirmation of the patient's consent was sent in writing to the patient including an information letter. Patients were made aware of their right to withdraw their consent at any time during or after the VC. Two patients subsequently withdrew their consent, leaving the total number of recordings at eight. Two female patients were not the actual subjects of the medical encounter but spoke on behalf of their children who were also present during the consultation. All consent forms – as well as the study itself – were approved by the institutional review board at the University of Southern Denmark, RIO (journal no. 11.052), in accordance with the GDPR and the Declaration of Helsinki.

Patients were selected by the GPs based on their willingness to participate rather than via specific selection criteria. Thus, participant recruitment took the form of convenience sampling.^33^ This sampling strategy was chosen as a pragmatic solution to assist patient recruitment. We acknowledged that consultations may constitute an event in which intimate health details and emotions are articulated, making patients less inclined to allow outsiders to witness – and much less record – their statements. Additionally, the implementation of VCs in Denmark in response to the COVID-19 pandemic was swift,^5^ possibly introducing uncertainties for both GPs and patients that stagnated the recruitment process. As data collection occurred in a general practice setting, the VCs include patients dealing with a variety of issues, related to both mental and physical conditions.

### Data analysis

All eight video recordings were imported directly into the CAQDAS programme NVivo 12 and were coded by the first author. Prior to this coding process, the recordings had been transcribed as part of an existing research project^34^ based on the GAT-2 conventions for minimal transcripts,^35^ strictly adhering to speech. Existing transcripts were used by the first author to cross-reference the interlocutors’ speech in instances of overlapping dialogue during coding. Additionally, notes on facial expressions and gestures were added to the transcripts and used in the subsequent coding process by the first author. The names of all patients have been pseudonymised for the purpose of this article.

Our analysis was guided by Braun and Clarke's *reflexive thematic analysis (RTA)*.^36–38^ The usefulness of applying RTA to video data to study concrete interactional phenomena through multiple modalities has been demonstrated by several studies.^39–41^ As afforded by the flexibility of RTA, our analytical process entailed moving on a continuum from inductive to deductive positions of reasoning.^38^ In the first phase, the recordings were viewed by the first author while writing down initial thoughts. In assessing the notes and returning to the data for internal validation, a patient empowerment (later participation) perspective was established, which became the unifying concept for the ensuing analysis. Upon reviewing the recordings through the established lens, Norman's concept of affordances^21^ was employed as a relevant analytical and interpretative framework. Subsequently, the second phase entailed a deductive coding of the data guided by patient participation and affordances, which drove the development of preliminary themes. Following joint group discussion and theme collation, the authors arrived at three final themes.

During the coding process, patients’ gaze directionality emerged as an area of interest. By watching the recordings in full, an approximation of the patient's smartphone screen could be made. Because of the smartphone's portable properties (a notion more thoroughly explained in the results), video-mediated communication often allows for a closer view of the speaker's face than would normally occur in face-to-face interactions. As such, the researchers had an up-close and clear view of the patients’ faces which allowed for the coding of gaze directionality by slowing down the recording and manually marking each shift of gaze away from the screen. Using this method, patients’ total on- and off-screen gaze durations could be assessed.

## Results

### Theme 1: Patient participation afforded by accessibility and audiovisuality

This theme highlights how the affordances of VCs allow consultation to occur despite spatial separation, ensuring participation on a fundamental – but important – level, as will be demonstrated with two examples.

During a consultation involving the patient Andrew and his GP, Andrew explains how he suffers from anxiety which often prevents him from venturing outside his home. He initially booked a physical consultation at the clinic, but due to an anxiety attack earlier that same day, the consultation was reorganised as a VC. Andrew explains that were it not for the reorganisation, he would most likely have failed to attend the consultation altogether because leaving his house induced too much stress on him. As such, it is specifically the potential for the VC to be conducted from the patient's home – in which such events can be avoided – that transformed the consultation from unfeasible to feasible. Here, the VC format made it possible for Andrew to still consult his GP by providing *accessibility* (a term coined by Islind et al.^26^). The provision of accessibility hinges on the technological capacities of smartphone devices to create a video-mediated point of contact between patient and GP. Moreover, being able to attend the consultation not only allows Andrew to participate in his health matters; he perceives attendance in itself as a step towards taking greater care of his mental health, as attending requires participation and active involvement: ‘just having a consultation with you [the GP] … it makes me get going. It makes me get into gear’ [08:23]. Therefore, participation through attendance is essential to Andrew in his ambition to manage his anxiety.

Another patient, Joan, had arranged a consultation for her child, who had recently suffered an allergic reaction. During the VC, Joan tells the GP that she herself is sick. Although she does not disclose the nature of her own illness, the illness is described as infectious to the point where she cannot send her child off to kindergarten and likewise – presumably – cannot attend an in-clinic examination with her child. In this respect, the VC format affords the consultation to be conducted immediately rather than being postponed, while simultaneously circumventing the risk of infection for the GP. Since the focus of this consultation is the assessment of the visual symptoms of the child's allergic reaction, the affordance of *audiovisuality* becomes an important component in providing accessibility. During Joan's initial description of her child's symptoms, the child is within the smartphone camera's field of view and thus available for the GP to assess visually in conjunction with Joan's verbal description. On several occasions during the consultation, Joan moves the camera closer to the child to assist in the GP's visual examination, for example, to demonstrate the swollen area around his eyes [00:33] and spots on his upper back [02:22]. The examination of the child is successful and leads to an agreement on a treatment plan. As such, participation is permitted through the affordances of accessibility and audiovisuality, rendering the VC a viable alternative to a physical consultation for these patients.

### Theme 2: Patient participation afforded by portability and audiovisuality

In the following, we describe how patients utilise resources in their immediate physical surroundings to participate in the ongoing interaction with their GP during their VC. To this end, the patients draw upon the affordances of portability and audiovisuality.

In the case of the patient Andrew, two distinct conditions enable him to move freely around his immediate surroundings without discontinuing the ongoing conversation with the GP. First, the VC app My Doctor anchors the consultation to Andrew's smartphone rather than to a computer screen or shared physical space such as the clinic. Second, the smartphone allows for the manipulation of the spatial placement of one's conversational partner; an affordance titled *portability* by Bardram and Houben^42^ and Schrock.^43^ Andrew utilises this affordance to maintain the conversation with his GP despite entering another room in his flat. During the VC, the GP asks: ‘You also take a small dose – as far as I know – of [medicine #1] seventy-five milligram, right?’ [11:00], to which Andrew responds with an ‘uhmm’ [11:04], grabs his smartphone from its placement and sets off to the medicine cabinet in his bathroom to check his medicine. By picking up his smartphone, we suggest that Andrew recognises a perceptual relationship between the portability of the device and his newly established goal of uncovering the dosage of his medicine. In doing this, the portability of the smartphone is actualised. As Andrew walks to his bathroom, the GP follows up his question with the assertion: ‘I should think so. It's what I’ve prescribed to you, at least’ [11:09]. This interaction is significant in terms of patient participation, as Andrew proceeds to disregard the GP's assertion of the validity of his own prescription. Andrew instead makes use of the opportunity to achieve certainty as afforded by the portability of his smartphone. Following a period of silence while Andrew walks towards the bathroom, the GP poses another question: ‘And then the [medicine #2] … do you take forty-five or thirty milligrams?’ [11:17]. Contrasting the preceding assertion, this question acknowledges the validity of the patient's intentions by encouraging him to pursue further clarification. During this sequence of interactions, we hold that the GP perceives the affordance of portability on which Andrew has already acted, which in turn shapes the subsequent pattern of communication between GP and patient. As the patient has autonomously chosen to pursue clarification of his medicine dosages despite the GPs assertion, the GP takes on the role of spectator and must await the patient's response before the consultation can progress further. Thus, the patient temporarily takes control of the consultation to achieve clarification regarding matters related to his health. The GP eventually encourages this by posing a supplementary question despite seemingly looking at the patient records himself.

Similarly, the patient Joan draws upon the affordance of audiovisuality to achieve clarification on her son's medication. She has used eye drops to treat her child's allergic reaction but reports little effect. During her report, a case of doubt arises between her and the GP related to the name of the eye drop medicine in question. Two communicative constraints may have triggered the case of doubt, the latter of which we will emphasise: firstly, it is clear from the patient's accent and her sentence construction that Danish is not her native language. Moreover, this particular VC exhibits a generally poor sound quality with frequent instances of distortion and latency-induced echoes. These types of technical deficiencies limit the effectiveness of auditory communication. As such, they constitute a constraint specific to the VC, as found by Björndell and Premberg^14^ and Sturesson and Groth.^44^ In this case, then, the VC's constraint coexists with its affordance of audiovisuality, both shaping the communicative bounds within which the patient-GP interaction plays out. As the GP reacts upon both emergent constraints, verbalisation does not constitute the primary communicative modality in the interaction but is supplemented by visually perceptible body language and gestures.

**Figure 1.**
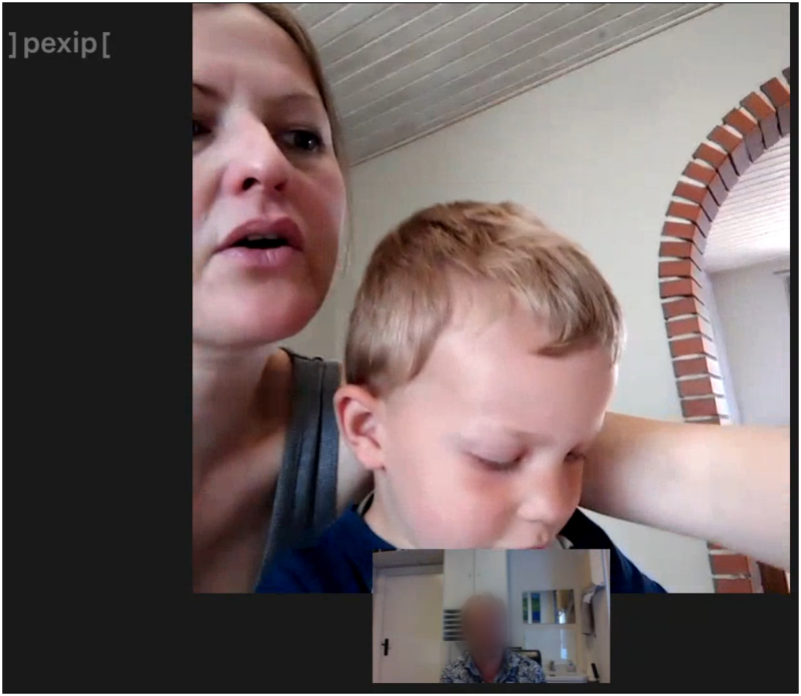
Joan reaches for the nearby medicine.

With the GP's ear turned towards the screen, evidently signalling his difficulty in understanding the patient, Joan states: ‘We only got the… Benaliv, or whatever it's called’ [00:44]. The GP immediately interrupts ‘Benadryl’ [00:46], correcting the patient. Joan then halts her sentence, orients her gaze away from the GP and brings into frame a small medicine bottle for the GP to see, hereby allowing him to deduce the correct name himself (Figure 1). Whether it is intentional or not, the patient counterbalances the auditory constraint experienced and signalled by the GP by prioritising the visual dimension of the VC in her concrete communicative act of describing the medicine in question. Thus, the patient is permitted to participate actively during the consultation by using resources present in her immediate surroundings. The GP, evidently able to discern the name of the medicine in question, exclaims ‘Yes’ [00:50], after which the bottle is put away. The patient then continues her report, having utilised visuality to establish a common point of reference.

### Theme 3: Patient participation signalled by portability and audiovisuality

Gaze direction provides important information for coordinating actions during social interaction.^45^ Existing research on interactions in video-mediated contexts indicates that VCs present constraints on the flow of such non-verbal communication.^46–48^ In this way, the VC's medium-specific characteristics sets the premises for how an interaction may play out, including how patients display participation. This theme illustrates how patients draw upon the VCs affordances of portability and audiovisuality as part of their non-verbal communication with their GP; signalling continued attention towards the ongoing conversation while *disengaging their gaze*^45^ from the smartphone to undertake deliberations during health-related inquiries initiated by the GP. As non-verbal communication is often unconscious,^49^ we do not view the actions of patients as deliberate or calculated, but rather as occurring intuitively in the context of the VC.

**Figure 2.**
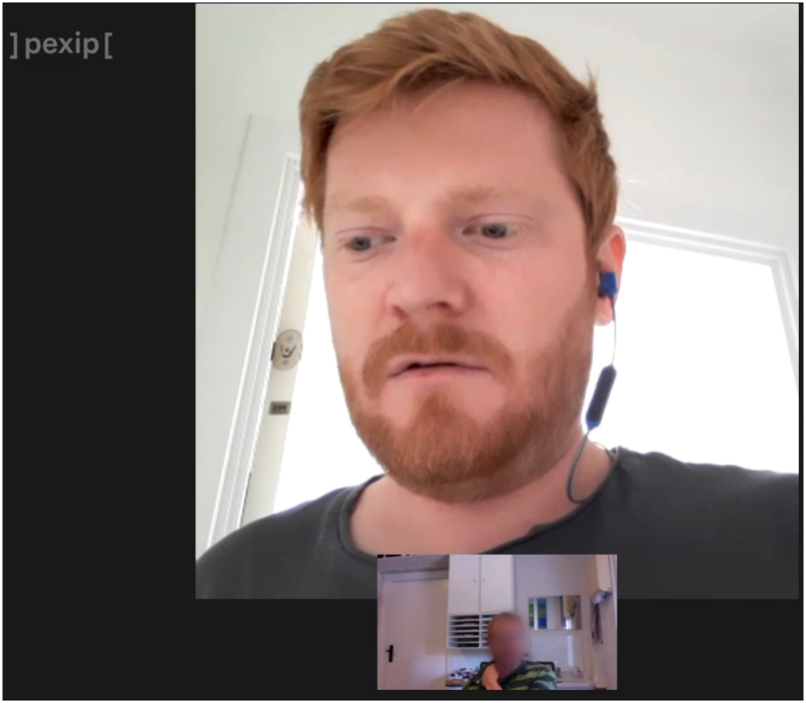
Andrew's gaze disengagement.

During their VCs, the patients Andrew and Thomas spend 53% and 40% of the time looking away from their smartphone and thus away from the GP. These gaze disengagements do not occur at random, but instead specifically in the wake of certain questions posed by the GP. After affirming his medicine doses (as described in theme 2), Andrew returns from the bathroom still carrying his smartphone. Based on the information provided by Andrew, the GP suggests an increase in his medicine dosage. Upon the GP's suggestion, Andrew immediately stops walking. At this moment, Andrews’ gaze is disengaged from the smartphone, directed down and to his right (Figure 2). Attempting a response, he cuts his sentence short: ‘Well, I would like to (sigh)…’ [11:48]. Of analytical significance is Andrews’ positioning of his smartphone in front of him, only slightly below head height. Although not addressing the GP by gaze, Andrew nonetheless has the smartphone camera angled towards himself, keeping his face and upper torso in frame. This marks a shift in position from when Andrew was walking, during which his smartphone was held around chest height with his face only partially visible. As the GP's view of the patient is determined by both the smartphone's capacity for audiovisuality and its positioning, the patient effectively controls the visual resources available to the GP. Thus, we argue that the change in position serves a communicative purpose related to Andrew's sudden pause and gaze disengagement. As gaze direction provides information for regulating and coordinating conversational flow,^50^ we suggest that Andrew here signals attention to the conversation by inviting the GP – albeit subconsciously – to perceive him as thinking attentively as a result of the GP's suggestion. Three observations from the scenario above form the basis of this suggestion: (1) the patient's attempt at responding, ending in a sigh signalling an end to his conversational turn, (2) the patient's gaze movement away from the VC and (3) the initial change of the patient's positioning of his smartphone to allow the GP to discern points 1 and 2. Further underlining this argument, the GP, seemingly correctly perceiving the patient as thinking but attentive, follows up on his initial suggestion by proposing an alternative treatment option while further elaborating on the reasons for his suggestion. After a subsequent period of silence lasting 2 seconds, the patient redirects his gaze towards the GP, visually signalling the end of his deliberations. He then articulates his preferred course of action.

Another patient, Thomas, is asked by the GP to account for his experiences during the treatment he had undergone to remedy his back pain. Throughout 89% of this account, which lasts 66 seconds [00:26–01:32], Thomas’ gaze moves between different focal points around the room in which he is sitting. Only after he concludes his report by accounting for his present state: ‘[i]t hurts like hell, actually’ [01:31], he returns his gaze to the VC on his smartphone to visually signal the end of his conversational turn. During his narrative, Thomas remains seated with his upper torso within the camera's field of viewand does not seem preoccupied with other tasks. By staying in frame, Thomas enables the GP to discern that his gaze disengagement is related to the task of recounting his treatment course rather than signalling a disengagement from the conversation. A similar incident occurs when Thomas is asked to recall the name of his rheumatologist [01:57]. Thomas disengages his gaze while turning his head away from the screen and upwards and to his left for a full 10 seconds before verbally responding. This action effectively halts the conversational flow. But by keeping his smartphone angled towards himself despite the significant head movement, Thomas’ non-verbal communication is available for the GP as a resource for interpreting his sudden head movement: he is engaged in recalling the requested name, and thus still attentive to the ongoing – albeit currently halted – conversation. This signalling takes place within the first 4 seconds of the 10-second recall period and is entirely based on visually mediated actions: gaze disengagement and smartphone positioning. The GP, his eyes focused upon the image of the patient, reacts to this visual signalling by repeating his question in a softer and quieter tone than the one used earlier. Only when prompted by the repeated question does the patient verbally contextualises his silence by uttering ‘eeehrm’ [02:02].

As demonstrated in the examples presented above, VCs put patients in control of visual resources when consulting the GP. The examples above illustrate how VCs constitute a communicative context wherein the affordances of audiovisuality and portability are intimately involved with coordinating interaction. Specifically, patients use these affordances to visually convey to the GP their continued attentional engagement despite being actively engaged in thought regarding either decision-making (Andrew) or recalling information (Thomas). As will be elaborated in the discussion section, displaying attention in this way may be able to enhance the patients’ ability to participate effectively during consultations, precisely because patients can visually convey their focus on deliberations about their health matters to the GP.

## Discussion

As indicated by our findings, it is important to consider even fundamental manifestations of participation in connection with VCs, such as enabling accessibility to health services. Echoing the findings of Christensen and Danbjørg^30^ and Assing Hvidt et al.,^51^ we found that VCs offered patients a convenient alternative to visiting the clinic. Additionally, one patient suffering from anxiety found the online format particularly accessible, as he struggled to leave his home. This is in line with existing research on telemental health solutions,^52,53^ in which patients suffering from anxiety perceived VCs as less intimidating than being in the room with the therapist, thus lowering the contact threshold. Indeed, the spatial settings in which medical consultations occur have been found by Chatwin et al.^54^ to create distinct atmospheres which influence some patients’ participation. Recognising that exposure to external stimuli increases cognitive load (an argument developed below), anxious patients may benefit from a reduction in such stimuli as anxiety disorders specifically affect cognitive behavioural performance.^55^ In substituting the GP's office with the patient's home and anchoring the conversation to the screen of their smartphone, VCs may present a viable option for this patient group.

Furthermore, we have illustrated that VCs enable patients to draw upon spatially situated resources for participatory ends. While Christensen and Danbjørg describe the patient's home as ‘almost sacred’.^30^^(p8)^ Islind et al. cite a nurse commenting on the VC as enabling a glimpse into the patient's ‘holiest place’.^26^^(p455)^ These perceptions demarcate the home as a setting in which patients can perform actions with a certain degree of confidence and immediacy. In a similar vein, Frittgen and Haltaufderheide^56^ emphasise that VCs conducted from the home enable patients to gain influence on the interaction, for instance by having contextual resources such as medicine or journals more readily available to them. As illustrated by our findings, patients draw upon the audiovisuality and portability of the VC and smartphone when utilising their surrounding resources. However, affordances remain culturally contingent^21^ and even vary between populations with similar cultures according to Oshlyansky et al.^57^ This invites us to reflect upon the context-specific implementation of video-mediated formats and their norms: how are spatial resources utilised in different settings (e.g., the workplace or hospitals), how suitable are VCs for different contexts (e.g., physical rehabilitation) and populations (e.g., patients living with anxiety)? To assess the value of VCs in these contexts, the findings reported in the present study should be more thoroughly explored by studies employing larger-scale designs.

In the meantime, we wish to situate our analysis of attentional engagement despite gaze disengagement among existing research literature illustrating how new technology introduces new patterns of communicative behaviour. Licoppe^58^ notes how mobile messaging affords short message bursts to reaffirm a sense of ongoing connected presence, while both Islind et al.^26^ and Nordtug et al.^59^ find that VC setups afford GPs’ division of attentive resources across different screens. Appropriating this premise that new technologies influences interaction,^48,60^ we consider VCs to constitute a context in which particular communicative behaviour may be employed. Similar to Benediktsson et al.'s^61^ finding that shifting surface-level displays of attention during smartphone conversations is not synonymous with conversational cessation, we found that gaze disengagement from the VC was contextualised by the patients' positioning of their smartphone. The communicative function of smartphone positioning pertains only to VCs, as this is the one consultation scenario where the patient controls the (audio)visual resources available to the GP for coordinating the interaction. Neither physical nor telephone consultations involve this type of control. We take this control to be significant in interpreting the patients’ recurring incidents of gaze disengagement when a question is posed by the GP, and its implications for the patients’ conversational participation in the consultation. Because of its relation to the GP's questions, we further posit that conveying gaze disengagement as active, thoughtful involvement in the interaction may enhance patients’ participation by allowing them to establish better rapport during the consultation. This suggestion builds upon the argument of Vredeveldt and Perfect^62^ that because doctors rely on patient reports to ensure appropriate treatment, allowing gaze disengagement may improve the patient's ability to accurately recollect information relevant for treatment. This is because by substituting the in-clinic consultation with a smartphone screen in the home, taxing environmental stimuli are reduced so that patients can direct their cognitive resources to better perform the task of recollecting or making decisions.^63–65^

Studies aiming to explore this line of reasoning may benefit from supplementing analyses of VCs with subsequent patient interviews to enrich their understanding of non-verbal behaviour. One ambition would be to construe such interviews as a form of member checking by testing the analytic interpretations made from the recordings against the patients’ own perceptions to heighten the credibility of the findings.^66^ However, such an endeavour may not be entirely unproblematic, as non-verbal communication is always continuous, but not always calculated: we can stop talking, but never stop communicating. Thus, retrospectively rationalising one's own non-verbal actions may be difficult. Rather, interviews could provide the researcher with more information about patients’ experiences of the VC in order to better contextualise the observed non-verbal behaviour and the interpretations derived hereof.

### Methodological reflections and limitations

A discussion of gaze during video-mediated interactions demands certain reflections, for example regarding latency and its effect on the interpretations made from the recordings. All VCs were recorded locally from the interface of the GP's computer. Thus, any connection issues or latency experienced by the GP would be captured by the recording software as well, perceptible as delayed or stuttering images or sound. Such instances are indeed observable during other VCs in the dataset but do not occur in the examples described in the results apart from Joan's VC. How reliably, then, can an analysis of gaze be conducted via recordings? One property of audiovisual recordings is the ability to slow down footage for a more granular analysis – a technique employed by several studies investigating gaze during social interactions.^67,68^ Despite its sensitivity to measurement error when compared with automated eye-tracking hardware, we argue that retroactively assessing gaze via manual coding allowed for more natural interactional patterns to occur during the VC as the patients were not required to wear any tracking equipment. However, we do not assume gaze patterns to be wholly predictable, as disengagement of gaze from one's conversational partner has previously been linked to different states such as social anxiety^69,70^ and uncertainty.^71^ Furthermore, the dynamics of gaze disengagement may differ across conversational topics, partners and settings. This has implications for the transferability of this study's findings to contexts outside VCs.

Although this study emphasises novel ways for patients to participate during VCs, it is pertinent to consider that other aspects are lost during a video-mediated patient-GP encounter. Although not prominent in the present study, significant latency may have implications for patient participation by hampering conversational flow. Technical deficiencies may, however, also inspire creative ways of interacting, as illustrated by the case of Joan in theme 2. Jepsen et al.^34^ contribute to such a nuanced understanding of VCs by showcasing how a throat examination may be performed despite the spatial separation of the patient and GP. Nonetheless, the loss of physical proximity holds implications for the scenarios in which VCs should be used, as the accuracy of the GP's assessment may be jeopardised by the inability to palpitate the patient. Sensory modalities such as smell or shortness of breath^31^ and subtle behaviour such as fidgety hands may also go unnoticed during VCs as a total impression of the patient's body may not be available to the GP. Likewise, the patient's full control of the visual resources gives rise to considerations concerning the distribution of responsibilities between patient and GP during VCs. This emphasises the need for collaborative efforts, in which the GP's attunement to VCs as a distinct communicative context is essential. In sum, GPs should continually assess the suitability of VCs in collaboration with the patient to avoid exacerbating these deficiencies, especially for those patients who primarily prefer VCs.

## Conclusion

As a medium, VCs comprise distinct affordances. By applying RTA to eight recorded VCs between patients and GPs and informed by the theoretical concept of patient participation, we developed three themes encapsulating different verbal and non-verbal modalities through which patient participation was enacted. Constituting a solution with high accessibility due to its internet-based connection of physically separated actors, VCs enabled patients to attend consultations with their GP despite being ill or – in the case of one patient – despite suffering from an anxiety attack precluding physical attendance. Moreover, patients drew on the portability and audiovisual capacity of their mobile devices to exercise influence on their interaction with the GP by utilising spatially contingent resources in their home. This assisted them in efficiently settling questions of doubt which arose during the consultation. Lastly, when drawing on portability and audiovisuality, patients contextualised their non-verbal communicative behaviour. They did this by displaying sustained attention towards the conversation with the GP while allowing for gaze disengagement when engaged in active decision-making or reporting with respect to matters related to their health. Thus, we find that VCs constitute a communicative context introducing distinct ways for patients to participate. More research is needed to fully explore the participatory opportunities of VCs in telemedical healthcare for different patient groups.
